# Highly efficient hyperentanglement concentration with two steps assisted by quantum swap gates

**DOI:** 10.1038/srep16444

**Published:** 2015-11-10

**Authors:** Bao-Cang Ren, Gui Lu Long

**Affiliations:** 1 State Key Laboratory of Low-Dimensional Quantum Physics and Department of Physics, Tsinghua University, Beijing 100084, China; 2 Tsinghua National Laboratory for Information Science and Technology, Beijing 100084, China; 3 Collaborative Innovation Center of Quantum Matter, Beijing 100084, P. R. China

## Abstract

We present a two-step hyperentanglement concentration protocol (hyper-ECP) for polarization-spatial hyperentangled Bell states based on the high-capacity character of hyperentanglement resorting to the swap gates, which is used to obtain maximally hyperentangled states from partially hyperentangled pure states in long-distance quantum communication. The swap gate, which is constructed with the giant optical circular birefringence (GOCB) of a diamond nitrogen-vacancy (NV) center embedded in a photonic crystal cavity, can be used to transfer the information in one degree of freedom (DOF) between photon systems. By transferring the useful information between hyperentangled photon pairs, more photon pairs in maximally hyperentangled state can be obtained in our hyper-ECP, and the success probability of the hyper-ECP is greatly improved. Moreover, we show that the high-fidelity quantum gate operations can be achieved by mapping the infidelities to heralded losses even in the weak coupling regime.

Entanglement has significant applications in quantum communication, such as quantum teleportation^1^, quantum dense coding^2^,^3^, quantum key distribution^4^,^5^, quantum secret sharing^6^, and quantum secure direct communication^7^,^8^. These tasks are carried out by distributing entangled photon pairs between the remote users. On one hand, an entangled photon system is produced locally and it suffers inevitably from its environment noise in its distribution process. On the other hand, the fiber attenuation is a challenge to be overcome with the exponential decrease of photon signals during transmission, which makes a photon be transmitted no more than several hundreds of kilometers. Quantum repeater is a current known approach to overcome this problem in long-distance quantum communication, while the entanglement of quantum systems decreases in the storage process as well. In this way, the fidelity and the security of long-distance quantum communication protocols will be decreased by decoherence. In order to improve the entanglement of quantum systems, entanglement purification and entanglement concentration are introduced in quantum repeaters.

Entanglement purification is introduced to extract fewer copies of nonlocal quantum systems in a high-fidelity entangled state from many noisy copies in a nonlocal less-entangled mixed state^9^,^10^,^11^,^12^,^13^,^14^, and entanglement concentration is used to distill fewer copies of nonlocal quantum systems in a maximally entangled state from many noisy copies in a nonlocal partially entangled pure state^15^,^16^,^17^,^18^,^19^,^20^,^21^,^22^,^23^,^24^. In 1996, Bennett *et al.*^15^ introduced the first entanglement concentration protocol (ECP) for improving the entanglement of partially entangled pure states with the Schmidt projection method. Many interesting ECPs have been proposed since this pioneering work. These ECPs can be divided into two groups. One group is proposed for a partially entangled pure state with its parameters unknown^16^,^17^,^18^,^19^,^20^,^21^,^22^ to the remote users, and the other group is proposed for a partially entangled pure state with its parameters accurately known^22^,^23^,^24^ to the remote users. In 2008, Sheng and Deng^18^ proposed a high-efficiency ECP for photon systems in a partially entangled Bell state by iterative application of the ECP process, resorting to nonlinear optical elements. This iteration protocol can also be used to improve the success probability of the ECPs for other entangled photon systems^14^,^19^.

Hyperentanglement, which is described as the quantum states entangled in different degrees of freedom (DOFs) of quantum systems^25^,^26^, is a promising resource with its fascinating applications in quantum computation (e.g., hyperparallel photonic quantum computation^27^) and quantum communication. With hyperentanglement, many quantum communication protocols have been proposed in a simple way, such as entanglement purification for polarization DOF of photon pairs^9^,^10^, complete Bell-state analysis^9^,^28^,^29^, and high-efficiency quantum repeater^30^. Also, there are many interesting long-distance high-capacity quantum communication protocols based on hyperentanglement, such as quantum teleportation^31^, entanglement swapping^31^,^32^, and hyperentangled Bell-state analysis^31^,^32^,^33^,^34^,^35^ based on the polarization and spatial-mode DOFs of photon systems. In 2008, Barreiro *et al.*^36^ demonstrated a superdense coding by using polarization-orbital-angular-momentum hyperentanglement, which has beaten the channel capacity limit with linear optics. In 2013, Ren *et al.*^22^ introduced the parameter-splitting method for concentrating the partially hyperentangled pure states with known parameters, which can obtain maximally hyperentangled states with the maximal success probability by using linear optical elements only, and they also proposed two hyperentanglement concentration protocols (hyper-ECPs) for the partially hyperentangled pure states with unknown parameters resorting to the Schmidt projection method^22^. By using the nonlinear optical elements, the success probability of the hyper-ECP with the Schmidt projection method can be improved by resorting to the iteration protocol^14^,^19^. However, these hyper-ECPs for photon systems were implemented by concentrating the polarization states and the spatial-mode states independently. In 2013, Vitelli *et al.*^37^ implemented experimentally the quantum-state-joining process for combining the two-dimensional quantum states of two input photons into an output single photon with linear optical elements.

In this article, we present a two-step hyper-ECP for nonlocal photon systems in polarization-spatial partially hyperentangled Bell states with the high-capacity character of hyperentanglement, resorting to the quantum swap gate for one DOF of photon systems. The swap gate is constructed with the giant optical circular birefringence (GOCB, defined as the differences in effective refractive index, phase, or reflection/transmission coefficients between the two circular polarizations^38^) of a nitrogen-vacancy (NV) center in a diamond embedded in the evanescent field of a photonic crystal cavity coupled to a waveguide (one-sided cavity-NV-center system), and it can be used to transfer the information in the polarization (spatial-mode) DOF between photon systems in the hyperentangled states. In the previous hyper-ECPs, the polarization states and the spatial-mode states are concentrated independently with polarization parity-check quantum nondemolition detector (P-QND) and spatial-mode parity-check quantum nondemolition detector (S-QND), respectively, where the success probability is limited without the information transfer within the hyperentangled photon pairs. In our two-step hyper-ECP, the swap gate is introduced to transfer the useful information between the partially hyperentangled photon pairs, so more photon pairs in the maximally hyperentangled state can be obtained, which has greatly improved the success probability (nearly equivalent to the success probability of ECP in one DOF). Moreover, our calculation shows that high-fidelity basic quantum gate operations can be achieved by mapping the infidelities to heralded losses even in the weak coupling regime. This two-step hyper-ECP with swap gates is very useful for obtaining maximally hyperentangled states in the long-distance high-capacity quantum communication protocols based on several DOFs of photon systems.

## Results

### Basic quantum gate elements for hyper-ECP

**Figure 1:**
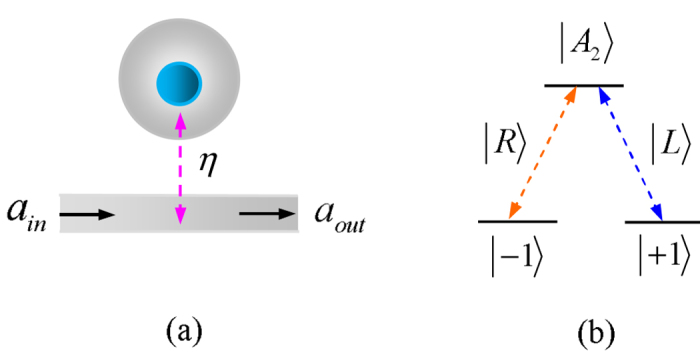
The GOCB of a diamond NV center embedded in the evanescent field of a photonic crystal cavity. (**a**) One-sided cavity-NV-center system, where the photonic crystal cavity is coupled to a waveguide. (**b**) The spin-preserving optical transition between the ground states 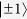 and the excited state 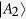 with the electronic orbital angular momentum change.

A cavity-NV-center system consists of a negatively charged NV center in diamond embedded in the evanescent field of a photonic crystal cavity, where the photonic crystal cavity is coupled to a waveguide as shown in Fig. 1(a). The negatively charged NV center is composed of a substitutional nitrogen atom, an adjacent vacancy, and six electrons. These six electrons come from the nitrogen atom and three carbon atoms surrounding the vacancy. The ground states of the negatively charged NV center are electronic spin triplet 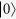 and 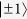 with a splitting of 2.88 GHZ, and their orbit states are 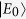. Here 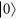 (*m*_*s*_ = 0) and 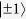 (*m*_*s*_ = ±1) are the magnetic sublevels, and the orbit state 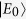 represents the angular momentum projection 0 along the NV axis. The excited states of the NV center are dependent of the Hamiltonian with the spin-orbit and spin-spin interactions and *C*_3*v*_ symmetry^39^. In the six excited states, the specifically excited state 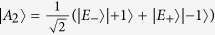 is robust with the stable symmetry^40^. Here, the orbit states 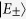 represent the angular momentum projections ±1 along the NV axis. In the spin-preserving condition, the optical transitions between the ground states and the excited states are created by the electronic orbital angular momentum change through the photon polarization. That is, if the NV center is in the ground state 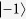 (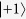), a right (left) circularly polarized photon 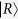 (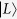) is absorbed to create the excited state 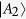 (shown in Fig. 1(b)).

The GOCB of a one-sided cavity-NV-center system can be calculated by the Heisenberg equations of motion for the cavity field operator 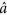 and diploe operator 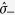
41,


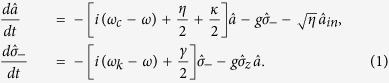


Here, *ω, ω*_*c*_, and *ω*_*k*_ (*k* = ±1) are the frequencies of the waveguide channel mode, the cavity field mode, and the energy transition between 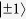 and 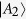, respectively. *η*/2 is the decay rate of the cavity field mode to the waveguide channel mode, and *κ*/2 is the decay rate of the cavity field mode to the cavity intrinsic loss mode. *γ*/2 is the decay rate of the dipole emitter in the NV center. *g* is the coupling strength between the cavity field mode and the dipole emitter in the NV center. 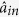 and 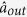 are the input and output field operators of the waveguide channel mode, and they are decided by the boundary relation 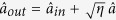. In the weak excitation limit with the NV center mainly in the ground state (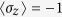), the reflection coefficient of the one-sided cavity-NV-center system can be expressed as^42^


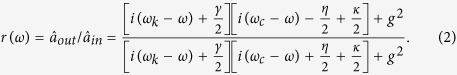


In the resonant condition *ω*_*c*_ = *ω*_*k*_ = *ω*, the reflection coefficient is 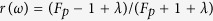 for *g* > 0, and it is 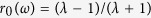 for *g* = 0. Here 
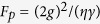
 is the Purcell factor, and 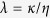. When 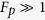, we have 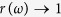. When the coupling strength is *g* = 0 and the cavity decay rate satisfies 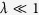, we have 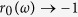. After the photon-spin interaction assisted by the cavity, the evolution of the states of the system composed of the photon and the electron spin in an NV center is expressed as:


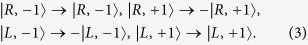


**Figure 2:**
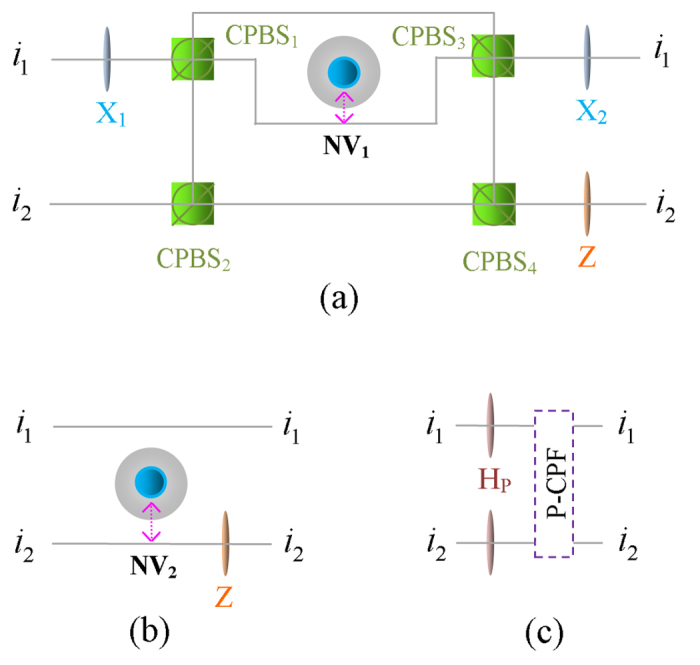
(**a**) Schematic diagram of the hybrid controlled-phase-flip gate for the polarization DOF of a photon (P-CPF). (**b**) Schematic diagram of the hybrid controlled-phase-flip gate for the spatial-mode DOF of a photon (S-CPF). (**c**) Schematic diagram of the swap gate for the polarization DOF of a two-photon system, resorting to a P-CPF gate. NV_1_ and NV_2_ are two one-sided cavity-NV-center systems. CPBS_*i*_ (*i* = 1, 2, …), the abbreviation of polarizing beam splitter in the circular basis, transmits the photon in the right-circular polarized state 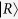 and reflects the photon in the left-circular polarized state 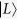, respectively. X_*i*_, which is implemented by a half-wave plate, performs a polarization bit-flip operation 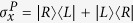 on a photon. Z performs a polarization phase-flip operation 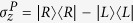 on a photon, and it is implemented by a half-wave plate. H_*P*_ performs a Hadamard operation on the polarization DOF of a photon [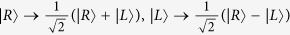], which can be implemented by a half-wave plate. *i*_1_ and *i*_2_ are the two spatial modes of photon *i* (*i* = *a*, *b*, *c*, *d*).

The basic gate elements of our hyper-ECP are constructed by the GOCB of the one-sided cavity-NV-center system, and their quantum circuits are shown in Fig. 2. The initial states of NV_1_ and NV_2_ are prepared in 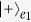 and 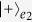, respectively. Here, 
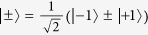
, and the states 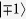 can be transformed into the superposition states 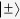 with a Hadamard operation, resorting to the microwave pulses^40^. That is, 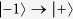 and 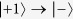. The two-photon system *AB* is initially in one of the partially hyperentangled Bell states 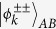 (*k* = 1, 2, 3, 4). Here, 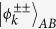 are polarization-spatial partially hyperentangled Bell states, and they are defined as


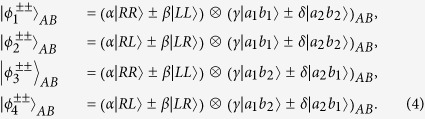


*i*_1_ and *i*_2_ are the two spatial modes of photon *i* (*i* = *a*, *b*).

#### Parity-check QND for the polarization DOF of two-photon systems

The parity-check QND for the polarization DOF of two-photon systems (P-QND) is used to distinguish the two-photon system with its polarization DOF in an even-parity mode from the one in an odd-parity mode, which is implemented with a hybrid controlled-phase-flip (CPF) gate for the polarization DOF of a photon. The setup of our hybrid CPF gate for the polarization DOF is shown in Fig. 2(a). Here, the initial state of photon *A* is 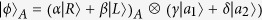. After we put the two wavepackets from spatial modes *a*_1_ and *a*_2_ of photon *A* into X_1_, CPBS (CPBS_1_ and CPBS_2_), NV_1_, CPBS (CPBS_3_ and CPBS_4_), X_2_, and Z in sequence, the state of the quantum system *Ae*_1_ can be transformed into


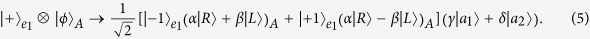


This is the result of the hybrid CPF gate, in which NV_1_ is used as the control qubit and the polarization DOF of photon *A* is used as the target qubit, without affecting the state of photon *A* in the spatial-mode DOF. We abbreviate this hybrid CPF gate as P-CPF.

If we have two photons *A* and *B* ( 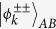 ) pass through the quantum circuit shown in Fig. 2(a) in sequence, the state of the system composed of NV_1_ and photon pair *AB* becomes


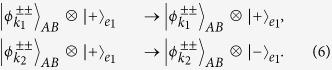


Here *k*_1_ = 1, 3 and *k*_2_ = 2, 4. The result of the P-QND can be obtained by measuring the electronic state of NV_1_ in the orthogonal basis 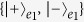. If the electronic state of NV_1_ is 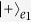, the polarization DOF of two-photon system *AB* is in an even-parity mode (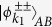). If the electronic state of NV_1_ is 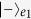, the polarization DOF of two-photon system *AB* is in an odd-parity mode (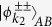).

#### Parity-check QND for the spatial-mode DOF of two-photon systems

The parity-check QND for the spatial-mode DOF of two-photon systems (S-QND) is used to distinguish the two-photon system with its spatial-mode DOF in an even-parity mode from the one in an odd-parity mode, which is implemented with a hybrid CPF gate for the spatial-mode DOF of a photon. The setup of our hybrid CPF gate for the spatial-mode DOF is shown in Fig. 2(b). If we let photon *A* in the state 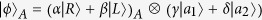 pass through NV_2_ and Z in sequence, the state of the quantum system 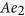 can be transformed into


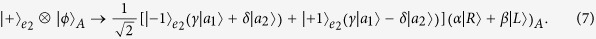


This is the result of the hybrid CPF gate, in which NV_2_ is used as the control qubit and the spatial-mode DOF of photon *A* is used as the target qubit, without affecting the state of photon *A* in the polarization DOF. We abbreviate this hybrid CPF gate as S-CPF.

If we have two photons *A* and *B* in the state 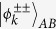 pass through the quantum circuit shown in Fig. 2(b) in sequence, the state of the system composed of NV_2_ and photon pair *AB* becomes


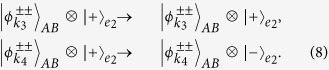


Here *k*_3_ = 1, 2 and *k*_4_ = 3, 4. The result of the S-QND can be obtained by measuring the electronic state of NV_2_ in the orthogonal basis 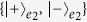. If the electronic state of NV_2_ is 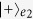, the spatial-mode DOF of two-photon system *AB* is in an even-parity mode (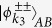). If the electronic state of NV_2_ is 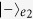, the spatial-mode DOF of two-photon system *AB* is in an odd-parity mode (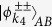).

#### Swap gate for one DOF of two-photon systems

Our swap gate is used to transfer the information in one DOF between photon systems encoded in both two DOFs. For example, the swap gate for the polarization (spatial-mode) DOF of two-photon system *AB* is used to swap the polarization (spatial-mode) states of photons *A* and *B*. The setup of our swap gate for the polarization DOF of a two-photon system is shown in Fig. 2(c), which is constructed with a P-CPF gate (shown in Fig. 2(a)). Suppose that the initial states of two photons *A* and *B* are


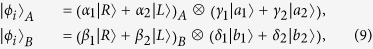


and the electronic state of NV_1_ is prepared in 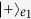.

We put two photons *A* and *B* into the quantum circuit shown in Fig. 2(c) in sequence, and the state of the system composed of photon pair *AB* and NV_1_ is transformed from 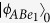 to 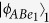. Here


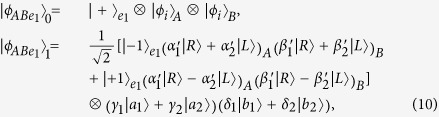


where 
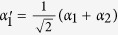
, 
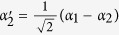
, 
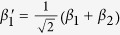
, and 
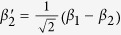
.

After the Hadamard operation is performed on the electronic state of NV_1_ we put two photons *A* and *B* into the quantum circuit shown in Fig. 2(c) again. These operations transform the state of the system composed of photon pair *AB* and NV_1_ from 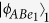 to 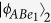. Here


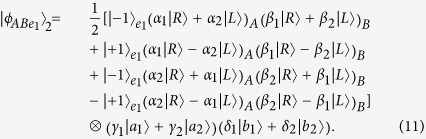


Finally, after another Hadamard operation is performed on the electronic state of NV_1_ again, the state of the system composed of photon pair *AB* and NV_1_ is transformed from 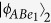 to 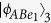. Here


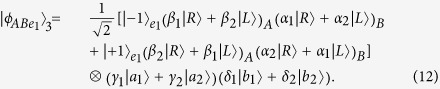


The result of the swap gate for the polarization DOF can be obtained by measuring the electronic state of NV_1_ in the orthogonal basis 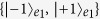 and performing condition operations on photons *A* and *B*. If the electronic state of NV_1_ is 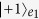, the bit-flip operations 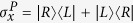 are performed on the polarization DOF of photons *A* and *B*. The final states of two photons *A* and *B* are


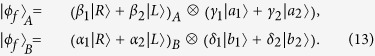


The swap gate for the spatial-mode DOF of two-photon systems can be constructed in the same way by replacing H_*p*_ and our P-CPF gate with BS and our S-CPF gate, respectively.

### Two-step hyper-ECP for partially hyperentangled Bell states

Our two-step hyper-ECP is used to distill some nonlocal photon pairs in maximally hyperentangled Bell state 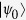 from those in partially hyperentangled Bell state 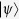 after the transmission over a noisy channel. Here


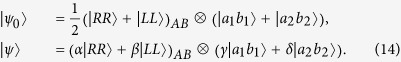


**Figure 3:**
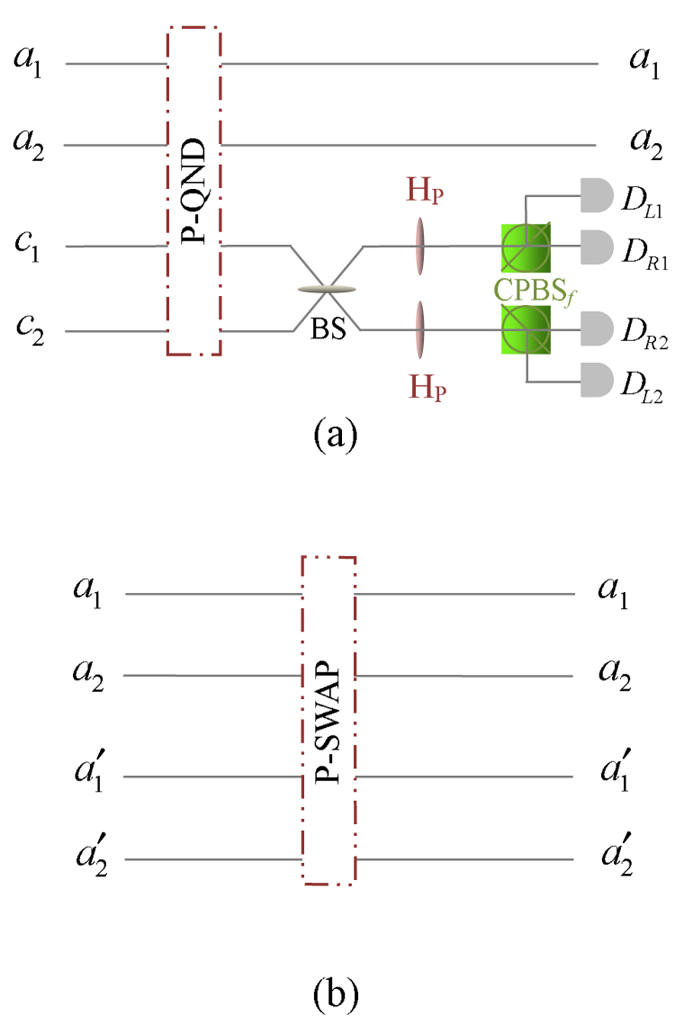
(**a**) Schematic diagram of the first step of our two-step hyper-ECP. The quantum circuit for Bob is the same as Alice by replacing P-QND and photons *A* and *C* with S-QND and photons *B* and *D*, respectively. (**b**) Schematic diagram of the second step of our two-step hyper-ECP. The quantum circuit for Bob is the same as Alice by replacing photons *A* and *A*′ with photons *B* and *B*′. *D*_*j*_ (*j* = *L*1, *R*1, *R*2, *L*2) is a single-photon detector. BS, the abbreviation of 50:50 beam splitter, performs a Hadamard operation on the spatial-mode DOF of a photon [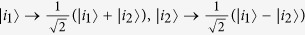]. P-SWAP (S-SWAP) is the swap gate for the polarization (spatial-mode) DOF of a two-photon system.

Now, let us introduce the principle of our two-step hyper-ECP, resorting to our quantum swap gate for one DOF. The setup of our two-step hyper-ECP with quantum swap gates is shown in Fig. 3. It includes two steps as shown in Fig. 3(a,b), and they are discussed in detail as follows.

#### The first step of our two-step hyper-ECP

In this step, we suppose that there are two identical two-photon systems in a nonlocal partially hyperentangled Bell state. That is,


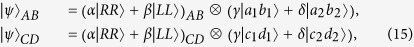


where the subscripts *AB* and *CD* represent two photon pairs. The two photons *A* and *C* belong to Alice, and the two photons *B* and *D* belong to Bob. *α, β, γ*, and *δ* are four unknown real parameters, and they satisfy the relation 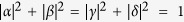.

The setup of the first step of our hyper-ECP is shown in Fig. 3(a). The initial state of four-photon system *ABCD* is 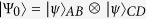. Alice performs the P-QND on photon pair *AC*, and Bob performs the S-QND on photon pair *BD*. After the measurements on the electronic states of the P-QND and S-QND, four cases will be obtained by Alice and Bob in this step.

(1) The outcome of the P-QND shows that the polarization DOF of photon pair *AC* is in an odd-parity mode, and the outcome of the S-QND shows that the spatial-mode DOF of photon pair *BD* is also in an odd-parity mode. In this case, the state of four-photon system *ABCD* is transformed into 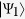 with the probability of 
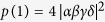
. Here


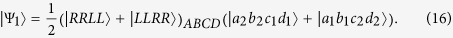


Subsequently, Alice performs the Hadamard operations on the polarization and spatial-mode DOFs of photon *C* as shown in Fig. 3(a), and Bob also performs the Hadamard operations on the polarization and spatial-mode DOFs of photon *D*. Then the state 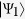 is transformed into 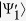. Here


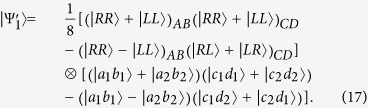


Finally, Alice and Bob detect photons *C* and *D* with single-photon detectors as shown in Fig. 3(a). If the outcome of the detection shows that the polarization DOF and the spatial-mode DOF of photon pair *CD* are both in the even-parity modes, the maximally hyperentangled Bell state 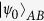 is obtained by Alice and Bob. If the outcome of the detection shows that the polarization DOF (the spatial-mode DOF) of photon pair *CD* is in an odd-parity mode, Bob has to perform the polarization phase-flip operation 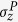 (the spatial-mode phase-flip operation 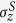) on photon *B* to obtain the state 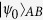. Here 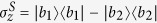.

(2) The outcome of the P-QND shows that the polarization DOF of photon pair *AC* is in an even-parity mode, and the outcome of the S-QND shows that the spatial-mode DOF of photon pair *BD* is also in an even-parity mode. In this time, the state of four-photon system *ABCD* is transformed into 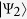 with the probability of 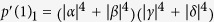. Here


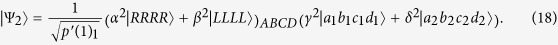


Subsequently, Alice and Bob perform the Hadamard operations and detections on two photons *C* and *D* as shown in Fig. 3(a), and the state 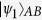 can be obtained after Bob performs the conditional local phase-flip operation 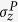 (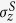) on photon *B*. Here the state 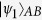 is a partially hyperentangled Bell state with less entanglement, and it is described as


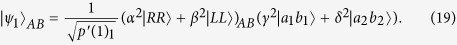


In this case, the polarization and spatial-mode DOFs of photon pair *AB* are both in partially entangled Bell states, so another round of our two-step hyper-ECP with quantum swap gates is required to obtain more nonlocal photon pairs in a maximally hyperentangled Bell state.

(3) The outcome of the P-QND shows that the polarization DOF of photon pair *AC* is in an even-parity mode, and the outcome of the S-QND shows that the spatial-mode DOF of photon pair *BD* is in an odd-parity mode. In this case, the state of four-photon system *ABCD* is transformed into 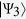 with the probability of 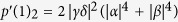. Here


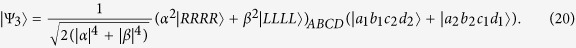


Subsequently, Alice and Bob perform the Hadamard operations and detections on two photons *C* and *D* as shown in Fig. 3(a), and the state 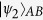 can be obtained after Bob performs the conditional local phase-flip operation 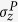 (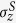) on photon *B*. Here


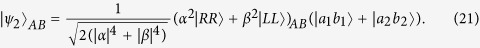


In this case, the spatial-mode DOF of photon pair *AB* is in a maximally entangled Bell state and the polarization DOF of photon pair *AB* is in a partially entangled Bell state with less entanglement, so the second step of our two-step hyper-ECP with quantum swap gates is required to transform the state of photon pair *AB* into a maximally hyperentangled Bell state.

(4) The outcome of the P-QND shows that the polarization DOF of photon pair *AC* is in an odd-parity mode, and the outcome of the S-QND shows that the spatial-mode DOF of photon pair *BD* is in an even-parity mode. Then the state of four-photon system *ABCD* is transformed into 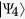 with the probability of 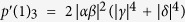. Here


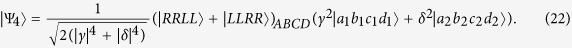


Subsequently, Alice and Bob perform the Hadamard operations and detections on two photons *C* and *D* as shown in Fig. 3(a), and the state 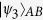 can be obtained after Bob performs the conditional local phase-flip operation 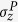 (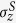) on photon *B*. Here


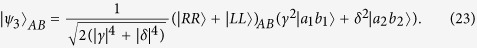


In this case, the polarization DOF of photon pair *AB* is in a maximally entangled Bell state and the spatial-mode DOF of photon pair *AB* is in a partially entangled Bell state with less entanglement, so the second step of our two-step hyper-ECP with quantum swap gates is required to transform the state of photon pair *AB* into a maximally hyperentangled Bell state.

#### The second step of our two-step hyper-ECP

In this step, the maximally hyperentangled Bell state 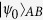 can be obtained from the cases (3) and (4) in the first step with our swap gates for one DOF, which can greatly improve the success probability of the hyper-ECP. The setup of the second step of our two-step hyper-ECP is shown in Fig. 3(b).

Suppose that there are another two identical two-photon systems 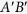 and 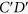, and they are in the states


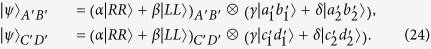


The two photons *A*′ and *C*′ belong to Alice, and the two photons *B*′ and *D*′ belong to Bob. In the first step, Alice and Bob perform the same operations on two-photon systems 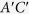 and 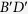 as they did on two-photon systems *AC* and *BD*.

If four-photon systems *ABCD* and *A*′*B*′*C*′*D*′ are projected into the states in the cases (3) and (4) in the first step, respectively, Alice and Bob can perform the polarization swap gates on two-photon systems 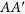 and 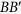 to transfer the useful information in the polarization DOF. In this way, the states of two-photon systems *AB* and 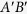 are transformed into 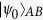 and 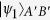 with the probability of 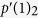 (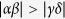).

If four-photon systems *ABCD* and *A*′*B*′*C*′*D*′ are projected into the states in the cases (4) and (3) in the first step, respectively, Alice and Bob can perform the spatial-mode swap gates on two-photon systems 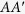 and 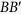 to transfer the useful information in the spatial-mode DOF. Then the states of two-photon systems *AB* and 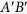 are transformed into 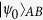 and 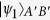 with the probability of 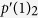 (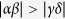).

At last, the state 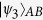 (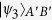) is left with the probability of 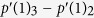 (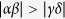) in this step. Another round of our two-step hyper-ECP with quantum swap gates is required for the two-photon systems in the states 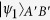, 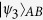, and 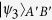 to obtain more nonlocal photon pairs in a maximally hyperentangled Bell state.

#### The success probability of our two-step hyper-ECP

After the first round of our two-step hyper-ECP, the success probability to obtain the maximally hyperentangled Bell state 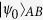 is 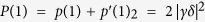 (for a pair of partially hyperentangled Bell states). The success probability of the hyper-ECP can be improved by iterative application of the two-step hyper-ECP process as discussed in the previous work^14^. For example, in the second round, Alice and Bob have to perform both of the two steps of this hyper-ECP on the photon pairs in the states 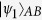 and 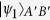, and they only have to perform the first step of this hyper-ECP on the photon pairs in the states 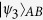 and 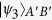. So the success probability of the states 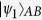 and 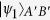 is 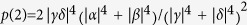, and the success probability of the states 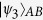 and 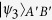 is 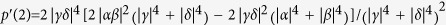. The success probability of the second round is 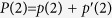 (for a pair of partially hyperentangled Bell states). The success probability of each round of our two-step hyper-ECP process is (for the case 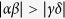)


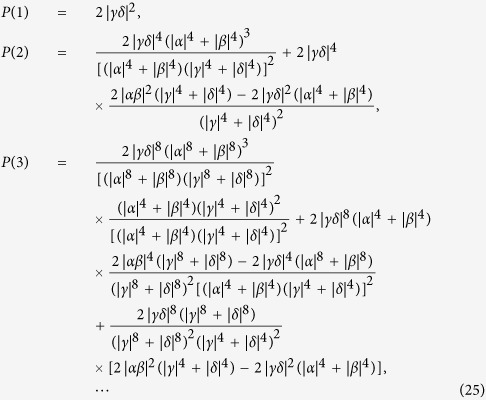


After *n* rounds of our two-step hyper-ECP process are completed, the entire success probability of the hyper-ECP is obtained as


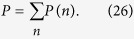


**Figure 4:**
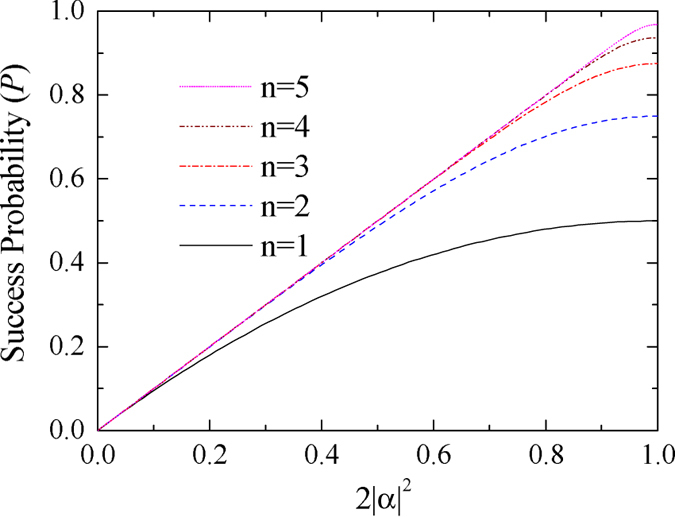
Success probability of our two-step hyper-ECP vs the parameter 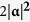 and the iteration number *n* (for a pair of partially hyperentangled Bell states). The parameters of the state 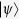 are 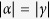 and 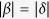.

In the ECP for quantum systems in one DOF^18^, the success probability of each round decreases exponentially with the increase of iteration number *n*. The success probability of each round of a hyper-ECP decreases much faster with the increase of iteration number *n* than the one of a ECP, when the polarization states and the spatial-mode states are concentrated independently. In the second step of our two-step hyper-ECP, swap gates are introduced to transfer the useful information between the photon pairs in partially hyperentangled Bell states, so more photon pairs in a maximally hyperentangled Bell state are obtained. This is different from the ECP for photon pairs in one DOF, because the information in one DOF can be transferred between the photon pairs in hyperentangled states, resorting to the high-capacity character of hyperentanglement. The success probability *P* vs the parameter 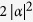 and the iteration number *n* is shown in Fig. 4 (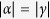). For instance, in the case 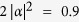 and *n* = 5, the success probabilities are 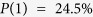 and 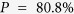 for the hyper-ECP without swap gates (the entire success probability *P* is nearly equivalent to the value 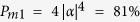)^14^. Here 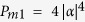 is the maximal value to obtain a maximally hyperentangled Bell state from a partially hyperentangled Bell state. In the same condition, the success probabilities are 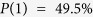 and 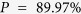 for our two-step hyper-ECP, where the entire success probability *P* is nearly equivalent to the value 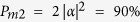. Here 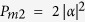 is the maximal value to obtain a maximally entangled Bell state from a partially entangled Bell state in one DOF. That is, the success probability of the hyper-ECP is greatly improved by transferring the useful information between the photon pairs in partially hyperentangled states, and the number of iteration steps is reduced in this condition.

## Discussion

### Fidelities of the basic gate elements

An NV center in diamond is a promising solid-state matter qubit for quantum information processing due to its long electron-spin decoherence time (~ms)^43^,^44^. With its long spin coherence time^45^,^46^ and nanosecond manipulation times^47^, an NV center in diamond can be used as a dipole emitter in the cavity QED to obtain the high-fidelity GOCB. There are many interesting works about NV centers in diamonds coupled to optical resonators (including optical cavities) both in theory^48^ and in experiment^49^,^50^,^51^,^52^,^53^. In experiment, the diamond NV center coupled to nanoresonator has been investigated either in the strong coupling regime^49^ or in the weak coupling regime^50^.

The quantum entanglement between the polarization of a single photon and the electron spin of an NV center in diamond is useful in quantum information network, which has been demonstrated in experiment^40^. If an NV center in diamond is coupled to a nanocavity, the spontaneous emission into the zero-phonon line can be greatly enhanced, which can improve the interaction between the NV center and the photon^51^,^52^,^53^. In 2012, Faraon *et al.*^51^ showed experimentally that the zero-phonon transition rate of an NV center can be greatly enhanced (~70) by coupling to a photonic crystal resonator (*Q* ~ 3000) fabricated in a monocrystalline diamond.

**Figure 5:**
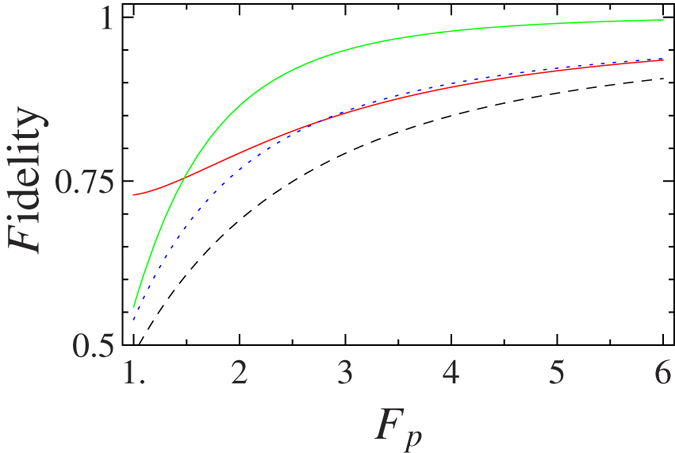
Fidelities of the basic gate elements. The green line represents the fidelity of the P-QND for the hyperentangled Bell state with its polarization DOF in an odd-parity mode (or the S-QND for the hyperentangled Bell state with its spatial-mode DOF in an odd-parity mode). The red line represents the fidelity of the P-QND (or the S-QND) for the hyperentangled Bell state with its polarization and spatial-mode DOFs both in the even-parity modes. The blue dotted line represents the fidelity of the P-QND for the hyperentangled Bell state with its polarization DOF in an even-parity mode and its spatial-mode DOF in an odd-parity mode (or the S-QND for the hyperentangled Bell state with its spatial-mode DOF in an even-parity mode and its polarization DOF in an odd-parity mode). The black dashed line represents the fidelity of the P-SWAP gate.

The reflection coefficients of the one-sided cavity-NV-center system are dependent of the Purcell factor *F*_*P*_ and the cavity decay rate *λ*. The fidelity of a quantum information process is defined as 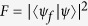, where 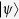 is the ideal final state of the quantum information process, and 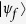 is the final state of the quantum information process in the experimental environment. The fidelities of our basic gate elements are shown in Fig. 5 with the cavity decay rate *λ* = 0.1^54^, and it shows that the fidelities are mainly reduced by the small Purcell factor *F*_*P*_. The fidelities of the basic gate elements may also be reduced by the large cavity decay rate *λ*^19^.

From Eq. (2), one can see that the reflection coefficients for *g* > 0 and *g* = 0 may be unequal (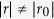), and it is 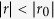 in experiment^55^,^56^. Here we show that the infidelities of the basic gate elements are mainly caused by 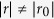, and 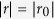 can be achieved by adjusting the Purcell factor *F*_*P*_ and the cavity decay rate *λ*. When the reflection coefficients are 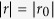, the Purcell factor *F*_*P*_ and the cavity decay rate *λ* should satisfy the relation 
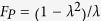
. In this time, the final states of the P-QND for hyperentangled Bell states are described as


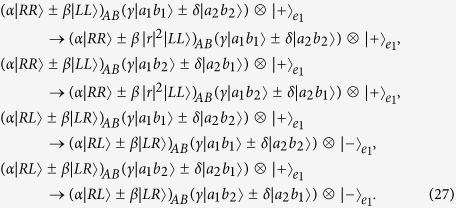


The final states of the S-QND for hyperentangled Bell states are described as


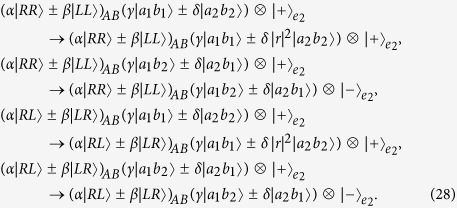


The final state of the P-CPF gate is described as


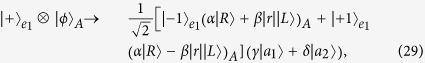


and the final state of the S-CPF gate is described as


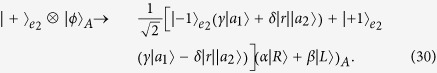


In Eqs. (27) and (28), the fidelities of the P-QND and S-QND are unit for the odd-parity modes, and the infidelities of the basic gate elements are transformed into the states of the photon systems in the case 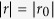. The infidelities of the photon systems can be transformed into the heralded loss by introducing the unbalanced BS (UBS) with the reflection coefficient 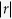 into the quantum circuits shown in Fig. 2(a,b), as introduced in the previous hyper-ECP^22^. For instance, with UBS, the P-CPF gate operation can be transformed into 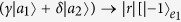

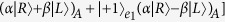

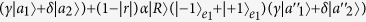
, and the infidelity of the P-CPF gate can be heralded if the photon is detected in the spatial modes 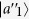 and 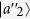, which is similar to the one introduced in the hyper-ECP with the parameter-splitting method^22^. That is, in the case 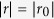, the infidelities of the basic gate elements can be mapped to the heralded loss^57^. If the cavity decay rate is adjusted to 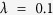, the Purcell factor is 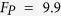 for 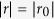, which requires 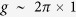 GHZ and 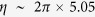 GHZ (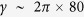 MHZ)^56^. The high-fidelity basic gate elements can be achieved even in the weak coupling regime, and the cavity intrinsic loss can be controlled in a appropriate regime instead of being set to zero for a high-fidelity quantum information processing, which may be easier to achieve in experiment.

In this hyper-ECP, the efficiency of the linear-optical elements and detectors, including PBSs, BSs, wave plates, and half-wave plates, are assumed to be perfect, which means there is no photon loss in linear-optical elements and detectors. In the practical application, the linear-optical elements and detectors may have inherent optical losses, so the success probability of each round of the hyper-ECP will be decreased. Because of the use of the swap gate, the success probability of each round of this hyper-ECP process is greatly improved, and the number of iteration steps is reduced, compared with the one without the swap gate. Hence the influence of the inherent optical losses is also reduced compared to that without the swap gate.

## Conclusion

We have presented a two-step hyper-ECP for polarization-spatial hyperentangled Bell states with the high-capacity character of hyperentanglement, resorting to the quantum swap gates for one DOF of photon systems. With the swap gate for one DOF of photon systems, the useful information can be transferred between the photon pairs in the hyperentangled states, so the success probability of each round of the hyper-ECP process is greatly improved. With our two-step hyper-ECP, more maximally hyperentangled Bell states are obtained and the number of iteration steps is reduced, compared with the one without the swap gate.

The basic quantum gate elements in our hyper-ECP, including P-QND, S-QND, and polarization (spatial-mode) swap gates, are constructed with the GOCB of one-sided cavity-NV-center systems. We showed that the high-fidelity basic gate elements can be achieved even in the weak coupling regime in the case 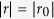, by mapping the infidelity to the heralded loss. Moreover, the cavity intrinsic loss can be controlled in a appropriate regime instead of being set to zero, and it may be easier to achieve in experiment.

By performing the swap gates on multiphoton system, our high-fidelity two-step hyper-ECP can be generalized for multiphoton hyperentangled states by transferring useful information between multiphoton systems in hyperentangled states. Besides hyper-ECP, the basic gate elements, including P-QND, S-QND, and polarization (spatial-mode) swap gates, can also be used to construct the high-efficiency hyperentanglement purification protocol for obtaining high-fidelity hyperentangled states from mixed hyperentangled states, by transferring useful information between nonlocal hyperentangled states. This will be the objective of a further work.

## Methods

### P-CPF gate

The setup of our P-CPF gate is shown in Fig. 2(a). The initial state of photon *A* is 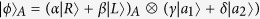. If we put the wavepacket from spatial mode *a*_1_ of photon *A* into X_1_, the state of photon *A* is changed to 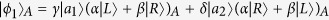. Subsequently, we put two wavepackets from spatial modes *a*_1_ and *a*_2_ of photon *A* into CPBS (CPBS_1_ and CPBS_2_), NV_1_, and CPBS (CPBS_3_ and CPBS_4_), and the state of the quantum system *Ae*_1_ is transformed into


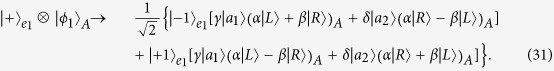


Finally, we put two wavepackets from spatial modes *a*_1_ and *a*_2_ of photon *A* into X_2_ and Z, and the state of the quantum system *Ae*_1_ is transformed into


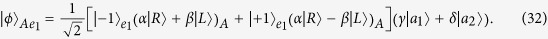


This is just the result of the P-CPF gate.

### S-CPF gate

The setup of our S-CPF gate is shown in Fig. 2(b). The state of photon *A* is prepared in 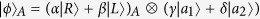. After the wavepacket from spatial mode *a*_2_ of photon *A* passes through NV_2_, the state of the quantum system *Ae*_2_ is transformed into


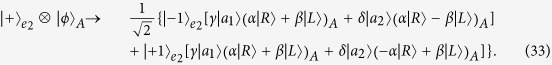


Subsequently, the wavepacket from spatial mode *a*_2_ of photon *A* is put into Z, and the state of the quantum system *Ae*_2_ is transformed into


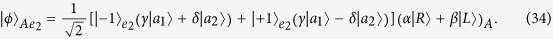


This is just the result of the S-CPF gate.

## Additional Information

**How to cite this article**: Ren, B.-C. and Long, G. L. Highly efficient hyperentanglement concentration with two steps assisted by quantum swap gates. *Sci. Rep.* **5**, 16444; doi: 10.1038/srep16444 (2015).
